# Can pretreatment lactate dehydrogenase to albumin ratio predict pathological complete response after neoadjuvant chemotherapy in breast cancer patients?

**DOI:** 10.5937/jomb0-43900

**Published:** 2025-03-21

**Authors:** Gözde Savaş, Nazan Günel, Ahmet Özet

**Affiliations:** 1 Gazi University, School of Medicine, Department of Medical Oncology, Ankara, Turkey

**Keywords:** LAR, LDH, albumin, neoadjuvant chemotherapy, breast cancer, complete response, LAR, LDH, albumin, neoadjuvantna hemoterapija, rak dojke, potpuna remisija

## Abstract

**Background:**

This study aims to evaluate the predictive significance of platelet lymphocyte ratio (PLR), neutrophillymphocyte ratio (NLR), lymphocyte monocyte ratio (LMR), systemic immune-inflammation (SII), prognostic nutritional index (PNI), haemoglobin, albumin, lymphocyte, and platelet (HALP) score and lactate dehydrogenase to albumin ratio (LAR) for pCR in breast cancer with neoadjuvant chemotherapy (NACT).

**Methods:**

A total of 121 patients who received NACT between February 2012 and November 2021 were included. LAR, NLR, PLR, MLR, SII, PNI and HALP were calculated using formulas. The cut-off value for markers was obtained by Receiver operating characteristic curve (ROC) analyses. Independent predictive factors for pCR were determined using multivariate regression analysis.

**Results:**

The pCR rate was achieved in 31.4% of patients. Median values of NLR, PLR, MLR, SII, PNI and HALP were similar in pCR (+) and pCR (-) (p>0.05). The median LAR value was significantly higher in pCR (+) than in pCR (-) (50.80 vs 42.62, respectively (p=0.002)). The optimal cut-off value of LAR was 46.27. Multivariate analysis showed that LAR ≥46.27 and HER-2 positivity were the independent predictive factors for pCR [OR=2.851 (95% CI=1.142-7.119, P=0.025), OR=3.431 (95% CI= 0.163-10.123, P=0.026), respectively].

**Conclusions:**

LAR is a simple, inexpensive, and convenient method for predicting pCR in breast cancer with NACT.

## Abstract (sr)

**Uvod:**

Ova studija ima za cilj da proceni prediktivni značaj odnosa trombocita i limfocita (PLR), odnosa neutrofila i limfocita (NLR), odnosa limfocita i monocita (LMR), sistemske imuno-inflamacije (SII), prognostičkog nutritivnog indeksa (PNI), skora hemoglobina, albumina, limfocita i trombocita (HALP) i odnosa laktat dehidrogenaze i albumina (LAR) za postizanje potpune patološke remisije (pCR) kod raka dojke uz neoadjuvantnu hemoterapiju (NACT).

**Metode:**

U istraživanje je uključeno ukupno 121 pacijentkinja koje su primile NACT u periodu od februara 2012. do novembra 2021. godine. LAR, NLR, PLR, MLR, SII, PNI i HALP su izračunati pomoću formula. Granitna vrednost markera određena je analizom ROC krive (Receiver Operating Characteristic curve). Nezavisni prediktivni faktori za pCR identifikovani su primenom multivarijantne regresione analize.

**Rezultati:**

Stopa pCR postignuta je kod 31,4% pacijentkinja. Srednje vrednosti NLR, PLR, MLR, SII, PNI i HALP bile su slične kod pacijentkinja sa pCR (+) i pCR (-) (p>0,05). Srednja vrednost LAR-a bila je značajno viša kod pacijentkinja sa pCR (+) nego kod onih sa pCR (-) (50,80 naspram 42,62, p=0,002). Optimalna granična vrednost LAR-a iznosila je 46,27. Multivarijantna analiza pokazala je da su LAR ≥46,27 i HER-2 pozitivnost nezavisni prediktivni faktori za pCR [OR=2,851 (95% CI=1,142-7,119, P=0,025), OR=3,431 (95% CI=0,163-10,123, P=0,026), redom].

**Zaključak:**

LAR predstavlja jednostavnu, jeftinu i praktičnu metodu za predikciju pCR kod raka dojke uz NACT.

## Introduction

Breast cancer is the most common cancer in women worldwide [1]. Neoadjuvant chemotherapy (NACT) was previously used to downstage locally advanced breast cancer or increase the rate of breast conservation. High-risk early-stage patients are also considered appropriate candidates for NACT [2]. Although NACT and adjuvant chemotherapy provide similar survival benefits, it has been shown that the prognosis is better in patients who achieve pathologic complete response (pCR) after NACT. Moreover, it is possible to tailor adjuvant therapy to subgroups such as HER-2 positive and triple negative subtypes without pCR [3] [4]. The risk of the occurrence of drugrelated side effects, disease progression risk until surgery, and postponement of surgery are the disadvantages of NACT. Therefore, it is essential to identify the patients who would benefit more from NACT. Though none has been validated, the molecular subtype, grade, and Ki-67 score are currently used to predict pCR. Many complex and expensive methods, such as genomic-proteomic classification, tumour-infiltrating lymphocytes (TILs) in the tumour, gene signatures, and ctDNA, have been proposed to increase predictive power. However, no validated method can currently be routinely used in clinical practice for predicting pCR in breast cancer [5].

Inflammation is closely associated with cancer formation and progression. It has been suggested that changes in neutrophils, lymphocytes, and platelets, which are peripheral indicators of inflammation, are related to the prognosis and survival of many cancers. Although there is more substantial evidence for associating platelet lymphocyte ratio (PLR), neutrophil- lymphocyte ratio (NLR) and lymphocytemonocyte ratio (LMR) with survival in breast cancer, there are conflicting results in terms of pCR [6] [7] [8]. A few studies have examined Systemic immune-inflammation (SII) in breast cancer with NACT [9].

Serum albumin level indicates nutritional status and is closely related to systemic inflammation. Malnutrition and inflammation decrease its synthesis. It has been shown that serum albumin levels are prognostic for breast cancer, and low albumin levels are associated with decreased survival [10] [11]. The prognostic nutrional index (PNI) and The haemoglobin, albumin, lymphocyte, and platelet (HALP) score are other indicators of nutritional status. The predictive roles of them have rarely been investigated in breast cancer patients with NACT [12] [13] [14].

Tumor cells meet their high-energy needs through glycolysis, in which lactate dehydrogenase (LDH) plays a role. LDH is involved in tumour cell proliferation and survival and could help evaluate tumour prognosis and treatment response [15]. It has previously been shown that LDH is a poor prognostic factor for metastatic and early breast cancer [11] [16]. No study has evaluated the LDH to albumin ratio (LAR) in breast cancer with NACT. The main objective of this study was to investigate the predictive ability of the inflammatory and nutritional biomarkers in breast cancer after NACT.

## Materials and methods

### Study population

This study included one hundred twenty-one eligible patients who received NACT in the Department of Medical Oncology of Gazi University between February 2012 and November 2021. The inclusion criteria were: (1) histologically confirmed breast cancer; (2) age ≥18 years; (3) having blood test results within 2 weeks before NACT; (4) undergoing surgery after NACT; (5) having accessible medical records. The exclusion criteria for the patients were: 1) with heart disease, liver disease, inflammatory disease, or active infection, (2) with other malignant tumours, and (3) received any oncological treatment before NACT. This retrospective cohort study was approved by the ethics committee of Gazi Medical School and designed following the Declaration of Helsinki.

### Data collection

The main clinicopathological characteristics (age, sex, Charlson Comorbidity Index(CCI), Eastern Cooperative Oncology Group (ECOG) performance score, histology, grade, lymphovascular invasion, stage, Ki-67); NACT type, pathologic and clinical response to NACT, and serum levels of neutrophil, monocyte, platelet, lymphocyte, haemoglobin, LDH and albumin were recorded retrospectively using patient files and hospital information system. LAR was calculated by dividing the serum LDH level by the serum albumin level. NLR was defined as the absolute neutrophil count divided by the absolute lymphocyte count, and PLR as the absolute platelet count divided by the absolute lymphocyte count. Systemic immune inflammation (SII), Prognostic nutritional index (PNI) and HALP score were calculated as follows: SII: neutrophil count × platelet count)/lymphocyte count; PNI: serum albumin (g/L) + 5 × total lymphocyte count (10^9^/L); HALP: hemoglobin (g/L) × albumin (g/L) levels × lymphocyte count (/L)/platelet count (/L). Estrogen receptor and progesterone receptor were evaluated immunohistochemically in pre-NACT biopsy samples, and positivity was determined as ≥1%. According to guidelines, HER-2 status was determined using immunohistochemistry and/or fluorescence in situ hybridization. Breast cancer subtypes were determined as luminal HER-2 (-), HER-2 enriched, and triple-negative. pCR was defined as the absence of residual cancer or carcinoma in situ in breast and axillary lymph nodes (ypT0ypN0) [17]. American Joint Committee on Cancer and Union for International Cancer Control TNM staging system was used for clinical staging [18].

### Statistical analysis

Statistical analyses were performed using SPSS version 25.0 (SPSS, Chicago, IL, USA). Histogram and Shapiro-Wilk tests were used to analyze the distribution of variables. Frequency, mean ± standard error, median, and interquartile range values were calculated according to the distribution characteristics of the variables. The chi-square test was used to compare categorical variables between the groups. Student’s t-tests or Mann-Whitney U tests were used to compare continuous variables according to distribution. Receiver operating characteristic (ROC) curve analysis was used to establish the optimal cut-off values of LAR, NLR, PLR, MLR, ALI, PNI, SIRI and HALP for the pCR prediction. Binary logistic regression analysis was performed to determine the contribution of multiple factors on pCR. p <0.05 was accepted as statistically significant.

## Results

### Characteristics of patients

The median age of the patients was 47 years (IQR: 40–59). According to clinical staging, 80 (66.1%) patients had T2, 28 patients (23.1%) had T4, 12 patients (9.9%) had T3, and 1 (0.8%) patient had T1. Only 2.5% of patients were N0, while 97.5% were lymph node-positive. Hormone receptor and HER-2 positivity rate were 70.2% and 22.3%, respectively. Anti-HER-2 targeted treatment was administered to all HER-2-positive cases. Of these patients, 102 (84.3%) were treated with anthracycline/taxan regimens, 10 (8.3%) with taxan-based regimens and 9 (7.4%) with anthracycline-based treatment. The patient characteristics are shown in Table 1.

**Table 1:** Baseline characteristics of patients. ECOG, Eastern Cooperative Oncology Group; CCI; Charlson Comorbidity Index, LVI: lymphovascular invasion; pCR, pathologic complete response; TNBC, triple negative breast cancer.

	(n=121)
Age, years (IQR)	47(40–59)
Sex (n, %) <br>Female <br>Male	<br>117(96.7) <br>4(3.3)
ECOG (n, %) <br>0<br>1	<br>96(79.3) <br>25(20.7)
CCI (n, %) <br>0<br>1	<br>86(71.1) <br>35(28.9)
Grade (n, %) <br>1<br>2<br>3	<br>10(8.3) <br>44(36.4) <br>67(55.4)
LVI (n, %) <br>yes <br>no	<br>40(33.1) <br>81(66.9)
Ki-67 (IQR)	40(20–70)
Stage (n, %) <br>2<br>3	<br>54(44.6) <br>67(55.4)
Histology <br>Invasive ductal carcinoma <br>Invasive lobular carcinoma <br>Others	<br>98(81.0) <br>5(4.1) <br>18(14.9)
Molecular subtype (n, %) <br>Hormone positive <br>Her2 positive <br>TNBC	<br>80(66.1) <br>27(22.3) <br>14(11.6)
pCR (n, %) <br>yes <br>no	<br>38(31.4) <br>83(68.6)

### Relationship between clinicopathological parameters and pCR

The pCR was achieved in 31.4% of patients. The patients were grouped according to pCR. The relationship between clinicopathological parameters and pCR is summarized in Table 2. HR negativity and HER-2 positivity were significantly higher in the pCR group (p<0.001 and p=0.033, respectively). The pCR was significantly higher in patients with Ki-67 ≥50 (p=0.001). The median values of LAR, NLR, PLR, MLR, ALI, PNI, SIRI and HALP according to pCR groups are shown in Table 3. The median LAR value was significantly higher in the pCR group (p=0.002).

**Table 2:** The relationship between pCR and clinicopathological characteristics of patients. ECOG, Eastern Cooperative Oncology Group; CCI, Charlson Comorbidity Index; LVI, lymphovascular invasion; TNBC: triple-negative breast cancer; ChT, chemotherapy.

	pCR+ <br>(n=38)	pCR- <br>(n=83)	p-value
**Age, years (IQR)**	46.50(40–57)	50(40–61)	0.555
**Sex (n, %) <br>**Female <br>Male	<br>37(97.4) <br>1(2.6)	<br>80(96.4) <br>3(3.6)	<br>1.000
**ECOG (n, %)** <br>0<br>1	<br>34(89.5) <br>4(10.5)	<br>62(74.7) <br>21(25.3)	<br>0.062
**CCI (n, %)** <br>0 <br>≥1	<br>29(76.3) <br>9(23.7)	<br>57(68.7) <br>26(31.3)	<br>0.390
**Grade (n, %)** <br>1<br>2<br>3	<br>2(5.3) <br>6(15.8) <br>30(78.9)	<br>8(9.6) <br>38(45.8) <br>37(44.6)	<br><br>**0.002**
**LVI (n, %) **<br>Yes <br>No	<br>13(34.2) <br>25(65.8)	<br>27(32.5) <br>56(67.5)	<br>0.855
**Ki-67 (IQR)**	50(30–80)	30(15–50)	**0.005**
Ki-67 (n, %) <br>≥50 <br><50	<br>22(57.9) <br>16(42.1)	<br>21(25.3) <br>62(74.7)	<br>**0.001**
**Stage (n, %) <br>**2<br>3	<br>13(34.2) <br>25(65.8)	<br>41(49.4) <br>42(50.6)	<br>0.119
**Molecular subtype (n, %)** <br>Hormone positive <br>HER-2 positive <br>TNBC	<br>15(39.5) <br>13(34.2) <br>10(26.3)	<br>65(78.3) <br>14(16.9) <br>4(4.8)	<br><br><**0.001**
**Hormon receptor status (n, %)** <br>Positive <br>Negative	<br>17(44.7) <br>21(55.3)	<br>68(81.9) <br>15(18.1)	**<br><0.001**
**Her-2 status (n, %)** <br>Positive <br>Negative	<br13(34.2) <br25(65.8)	<br14(16.9) <br69(83.1)	<br>**0.033**
**ChT (n, %) <br> **Antracyclin <br>Taxan <br>Antracyclin+taxan	<br>2(5.3) <br>4(10.5) <br>32(84.2)	<br>7(8.4) <br>6(7.2) <br>70(84.3)	<br><br>0.726

**Table 3:** The association between pCR and LAR, NLR, PLR, LMR, PNI, SII, HALP. pCR, pathologic complete response; LAR, lactate dehydrogenase to albumin ratio; NLR. neutrophil-lymphocyte ratio; PLR, platelet lymphocyte ratio; LMR. lymphocyte monocyte ratio; PNI, prognostic nutritional index; SII. systemic immune-inflammation; HALP, haemoglobin, albumin, lymphocyte, and platelet.

	pCR + <br>(n=38)	pCR + <br>(n=38)	p-value
LAR (±SD)	53.52+16.34	44.36+10.16	**0.002**
NLR (±SD)	2.70±2.29	2.60±1.39	0.843
PLR (±SD)	157.54±77.54	152.31±69.50	0.712
LMR (±SD)	4.08±2.68	4.10±1.54	0.962
PNI (±SD)	42.91	43.91±3.65	0.202
SIRI (±SD)	1.59±1.51	1.52±1.14	0.752
HALP (±SD)	42.42±19.95	47.52±33.79	0.390

**Figure 1:**
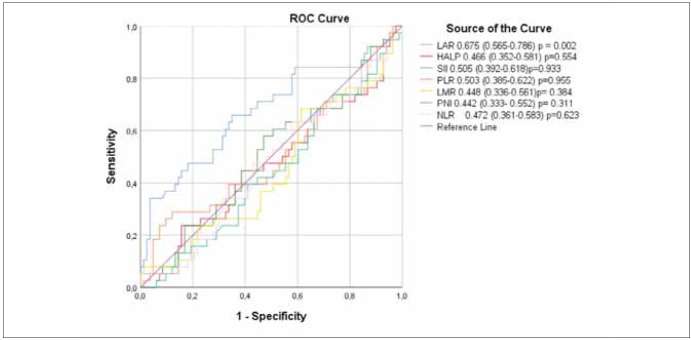
Predictive performances of the pretreatment LAR, HALP, SII, PLR, LMR, PNI, NLR by ROC curve analysis. LAR, lactate dehydrogenase to albumin ratio; HALP, haemoglobin, albumin, lymphocyte, and platelet score; SII, systemic immuneinflammation; PLR, platelet lymphocyte ratio; LMR: lymphocyte monocyte ratio; PNI, prognostic nutritional index; NLR, neutrophil lymphocyte ratio.

The optimal cut-off values of LAR, NLR, PLR, MLR, ALI, PNI, SIRI and HALP were determined using ROC curve analysis. The optimal cut-off of LAR was 46.27 (Area under the curve [AUC], 0.675; 95% Confidence intervals [CI] 0.565–786, p=0.002) with a sensitivity of 65.8% and a specificity of 65.1%. The ROC analysis is presented in Figure 1.

The independent predictive factors for pCR were explored using univariate and multivariate logistic regression analyses. Based on these analyses, it was determined that higher LAR (odds ratio [OR], 2.851; 95% CI, 1.142–7.119, p=0.025) and HER-2 positivity (OR, 3.431; 95% CI, 1.163–10.123, p=0.026) were independent prognostic factors for pCR (Table 4).

**Table 4:** Multivariate logistic regression analysis of factors predicting pathologic complete response. LAR, LDH to albumin ratio; HR, hormone receptor

LAR ( ≥46.27 vs <46.27)	2.851	1.142–7.119	**0.025**
HR (Negative vs positive)	2.669	0.994–7.170	0.051
HER-2 (Positive vs negative)	3.431	1.163–10.123	**0.026**
Grade (3 vs 1–2)	2.421	0.796–7.367	0.119
Ki-67 (≥50% vs <50%)	2.734	0.909–8.219	0.073

## Discussion

Currently, NACT is recommended as a standard treatment modality in the management of locally advanced breast cancer, early-stage patients who are not suitable for breast-conserving surgery, and HER- 2 positive or triple negative subgroups with tumours ≥2 cm [2]. pCR after NACT is considered a surrogate marker for prolonged survival (disease-free and overall survival) [3] [4]. The relationship between pCR and survival is more pronounced for HER-2 positive and triple negative subgroups, but it is also true for luminal B tumours. However, the pCR rates remained low in these subgroups. (12% for luminal B / HER2 −, 30–40% for TN, 30–50% for HER2+) [17]. Therefore, determining which patients can achieve pCR after NACT is critical for appropriate patient selection. No validated method is available for use in routine practice to predict pCR. Currently identified molecular subtypes of breast cancer are insufficient for predicting pCR. Recent studies have revealed that genomic and transcriptomic breast cancer subtypes have different clinical courses, which may shed light on the selection of the most suitable candidates for NACT [18]. Although studies show the relationship between TILs and pCR in TNBC, they have not yet been used in daily practice [19]. Many complicated and expensive methods, such as circulating tumour DNA and gene signature imaging, are still under investigation [5]. This study examined the relationship between inflammatory markers and pCR in breast cancer with NACT. While no significant relationship was found between NLR, PLR, MLR, ALI, PNI, SIRI, HALP and pCR, high LAR was significantly associated with pCR. Moreover, only LAR was found to be an independent factor in pCR.

It has been shown that inflammation takes part in all stages of tumorigenesis. The prognostic significance of peripheral inflammation markers has been investigated in different cancer types and breast cancer. It has been shown that pre-NAC PLR, NLR and LMR can predict pCR. However, as the available studies are not prospectively designed and include heterogeneous patient groups, the exact relationship between inflammatory markers and pCR could not be determined [19]. A recent meta-analysis examined the relationship between longitudinal change in NLR (delta-NLR) and chemotherapy response in patients who underwent NACT. While delta-NLR was stable in cases with pCR, an increase in NLR was detected in the non-pCR group. It has been suggested that dynamic changes in the NLR during NACT are more predictive for pCR [20]. We could not find any association between NLR, PLR, LMR and pCR. Potential reasons for this discrepancy may be the inclusion of all molecular subtypes of breast cancer and the heterogeneity of NACT types in our study. In addition, the definition of pCR as the absence of both invasive tumour and ductal carcinoma in situ may also impact the results.

SII is considered a more inclusive inflammatory marker due to the contribution of monocytes. It was previously determined that SII was associated with breast cancer prognosis [21]. However, the relationship between SII and pCR is still unclear. In the study by Dong et al. [9], which included 241 patients with breast cancer receiving NACT, low SII was found to be predictive for pCR. In contrast, in the study by Chen et al. [22], no relationship was found between SII and pCR. Similarly, we found that SII was not associated with pCR. The heterogeneity of existing studies may explain these discrepancies.

HALP is an indicator of inflammatory and nutritional status. As far as we know, two studies in the literature evaluate the relationship between pCR and HALP in breast cancer. Lou et al. [13] showed that patients with HALP >24.14 had a higher pCR rate in TNBC [13]. In contrast, in the study of Yüce et al. [12], which included 127 breast cancer patients who underwent NACT, no significant relationship was found between HALP and pCR. Similarly, we could not find any correlation between the HALP score and pCR. Conflicting results have been obtained in the limited number of studies evaluating the relationship between NACT response and PNI. In our study, no significant relationship was found between PNI and pCR. More comprehensive studies are needed to clarify this uncertainty.

Cancer cells use anaerobic glycolysis to provide energy regardless of oxygen availability [23]. LDH catalyzes the reversible conversion of pyruvate to lactate in glycolysis. LDH participates in tumour formation, invasion, recurrence, and metastasis [24]. Many studies have been conducted on the prognostic value of serum LDH levels in breast cancer, with a meta-analysis showing it to be prognostic in terms of survival [25]. Albumin is a protein that regulates oncotic pressure and plays a role in substance transport (ligands, drugs, etc.). It is considered an indicator of nutritional status, and malnutrition and inflammation decrease its synthesis. Low albumin level is an independent poor prognostic factor for survival in early and advanced breast cancer [11]. LAR is calculated as the ratio of serum LDH level to serum albumin level, which can indicate the systemic inflammatory status. LAR has previously been shown to be a poor prognostic factor for survival in colon carcinoma, hepatocellular carcinoma, and pancreatic cancer [26] [27] [28]. To our knowledge, the association between LAR and pCR has not been evaluated in patients with breast cancer receiving NACT. The results of our study showed that high LAR values can predict pCR. The LAR value was determined to be higher in the triple-negative and HER-2 positive subgroups, which had a higher chance of achieving pCR after NACT. However, multivariate analysis revealed that HER-2 positivity and high LAR significantly predicted pCR.

The study’s major strength was that it was the first report to show the relationship between pCR and LAR. The limitations of our study were its retrospective nature, relatively small sample size and heterogeneity of patient selection. However, this is a unique study evaluating the relationship between pCR and LAR in breast cancer with NACT. The ability of LAR to predict pCR even in a heterogeneous group of breast cancer patients makes it useful in daily practice.

## Conclusion

This study indicates that high LAR significantly predicts pCR in breast cancer patients with NACT. As a result, LAR, an easily accessible and inexpensive parameter, can help identify patient groups that will benefit most from NACT and guide the treatment strategies. The precise relationship between pCR and LAR should be elucidated in future prospective studies.

## Dodatak

### Conflict of interest statement

All the authors declare that they have no conflict of interest in this work.
